# Genetic and environmental risks for clonal hematopoiesis and cancer

**DOI:** 10.1084/jem.20230931

**Published:** 2024-12-03

**Authors:** Stephanie Franco, Lucy A. Godley

**Affiliations:** 1 Department of Medicine, Northwestern Medicine, Chicago, IL, USA; 2 Division of Hematology/Oncology, Department of Medicine, Robert H. Lurie Comprehensive Cancer Center, Feinberg School of Medicine, Northwestern University, Chicago, IL, USA

## Abstract

Somatic variants accumulate in all organs with age, with a positive selection of clonal populations that provide a fitness advantage during times of heightened cellular stress leading to clonal expansion. Easily measured within the hematopoietic compartment, clonal hematopoiesis (CH) is now recognized as a common process in which hematopoietic clones with somatic variants associated with hematopoietic neoplasms exist within the blood or bone marrow of individuals without evidence of malignancy. Most cases of CH involve a limited number of genes, most commonly *DNMT3A*, *TET2*, and *ASXL1*. CH confers risk for solid and hematopoietic malignancies as well as cardiovascular and numerous inflammatory diseases and offers opportunities for cancer prevention. Here, we explore the genetic and environmental factors that predispose individuals to CH with unique variant signatures and discuss how CH drives cancer progression with the goals of improving individual cancer risk stratification, identifying key intervention opportunities, and understanding how CH impacts therapeutic strategies and outcomes.

## Teaser

This article explains how germline genetic factors and environmental exposures shape clonal hematopoiesis, the accumulation of somatic variants within hematopoietic tissues during one’s lifespan, which confers risk for solid and hematopoietic malignancies as well as cardiovascular and numerous inflammatory diseases.

## Introduction

Somatic variants within hematopoietic stem and progenitor cells (HSPCs) occur as a result of random mitotic errors and subsequent DNA damage (Kessler et al., 2022). The persistence of these errors may result from the failure of DNA damage repair mechanisms, such as double-strand break repair via homologous recombination. These somatic variants accumulate with age and often do not confer major significance to cells, resulting in increased genetic heterogeneity, a form of somatic mosaicism (Quiros and Vassiliou, 2023; Vijg and Dong, 2020). When somatic variants provide a fitness advantage through enhanced survival and/or proliferation, positive selection and expansion of clonal populations may arise during times of heightened cellular stress (Kessler et al., 2022; Xie et al., 2014). Selection and expansion of clonal populations containing somatic variants that possess a survival advantage can lead eventually to malignant transformation (Bolton et al., 2020). Clonal hematopoiesis (CH) refers to the existence of clonal populations of hematopoietic cells containing somatic variants associated with myeloid neoplasms found in the blood or bone marrow of individuals without other evidence of hematopoietic malignancy (Singh and Balasubramanian, 2024). Less commonly, somatic variants in genes associated with lymphoid malignancies are also involved (Quiros and Vassiliou, 2023). Drivers of CH can include any somatic variant, ranging from single nucleotide variants (SNVs) and insertions/deletions to large-scale chromosomal alterations (most commonly, loss of the Y chromosome) (Evans and Walsh, 2023). CH in the absence of a known driver gene variant is likely associated with mosaic chromosome alterations, which also increase in prevalence with age (Evans and Walsh, 2023). Evans and Walsh provide a more detailed overview of CH associated with chromosome alterations in their review of CH and somatic mosaicism (Evans and Walsh, 2023).

Generally, clones with a variant allele frequency (VAF) ≥2% are considered to be of potential significance, with the median VAF being ∼16% (Heuser et al., 2016; Bick et al., 2020). Clonal hematopoiesis of indeterminant potential (CHIP) refers to CH involving genes associated with hematopoietic malignancy with a VAF of at least 2% (Craven and Ewalt, 2023). Clonal cytopenia of undetermined significance (CCUS) refers to somatic variants associated with otherwise unexplained cytopenias and is generally considered to be a direct precursor of myelodysplastic syndrome (MDS) or acute myeloid leukemia (AML) (Craven and Ewalt, 2023).

A relatively limited number of genes comprise most cases of CH. Studies report that more than 75% of cases of CH are associated with *DNMT3A*, *TET2*, and *ASXL1* variants, and 15% are associated with the next five most frequent genes: *PPM1D*, *JAK2*, *SF3B1*, *SRSF2*, and *TP53* (Bick et al., 2020). The genes most commonly associated with CH can be categorized as epigenetic regulators (e.g., *DNMT3A*, *TET2*, and *ASXL1*), DDR genes (e.g., *PPM1D*, *TP53*, *CHEK2*, and *ATM*), cellular growth signals (*JAK2*), and spliceosome genes (*SF3B1* and *SRSF2*) (Joo et al., 2023). CH has been implicated in the pathogenesis of cardiovascular and other inflammatory diseases, primary solid and hematopoietic malignancies, and therapy-related myeloid neoplasms (t-MNs), and is consequently associated with increased all-cause mortality (Kessler et al., 2022). Interestingly, the various somatic variants associated with CH result in heterogenous phenotypes with their own clinical consequences—for example, clones with *DNTMT3A* variants are most strongly associated with malignancy, whereas those involving *JAK2* are associated with the highest degree of cardiovascular disease (Kessler et al., 2022).

Recognition of CH as a risk factor for de novo and treatment-related malignancies offers several potential opportunities for intervention prior to the development of aggressive and often difficult-to-treat cancers. Although excellent reviews of the role of CH in the pathogenesis of t-MNs have been published recently (Travaglini et al., 2024), here we provide a complementary review in which we place the development of CH within the broader context of individual cancer risk over one’s lifespan, beginning prior to its development, and exploring the genetic and environmental factors that predispose individuals to acquisition of somatic variants associated with CH, the unique signature of these variants, and the drivers of progression to malignant disease. With this understanding, we can characterize individual cancer risk, identify key opportunities for intervention, and consider the impact on potential therapies.

## Risk factors for CH

### Germline genetics

**Figure 1.**
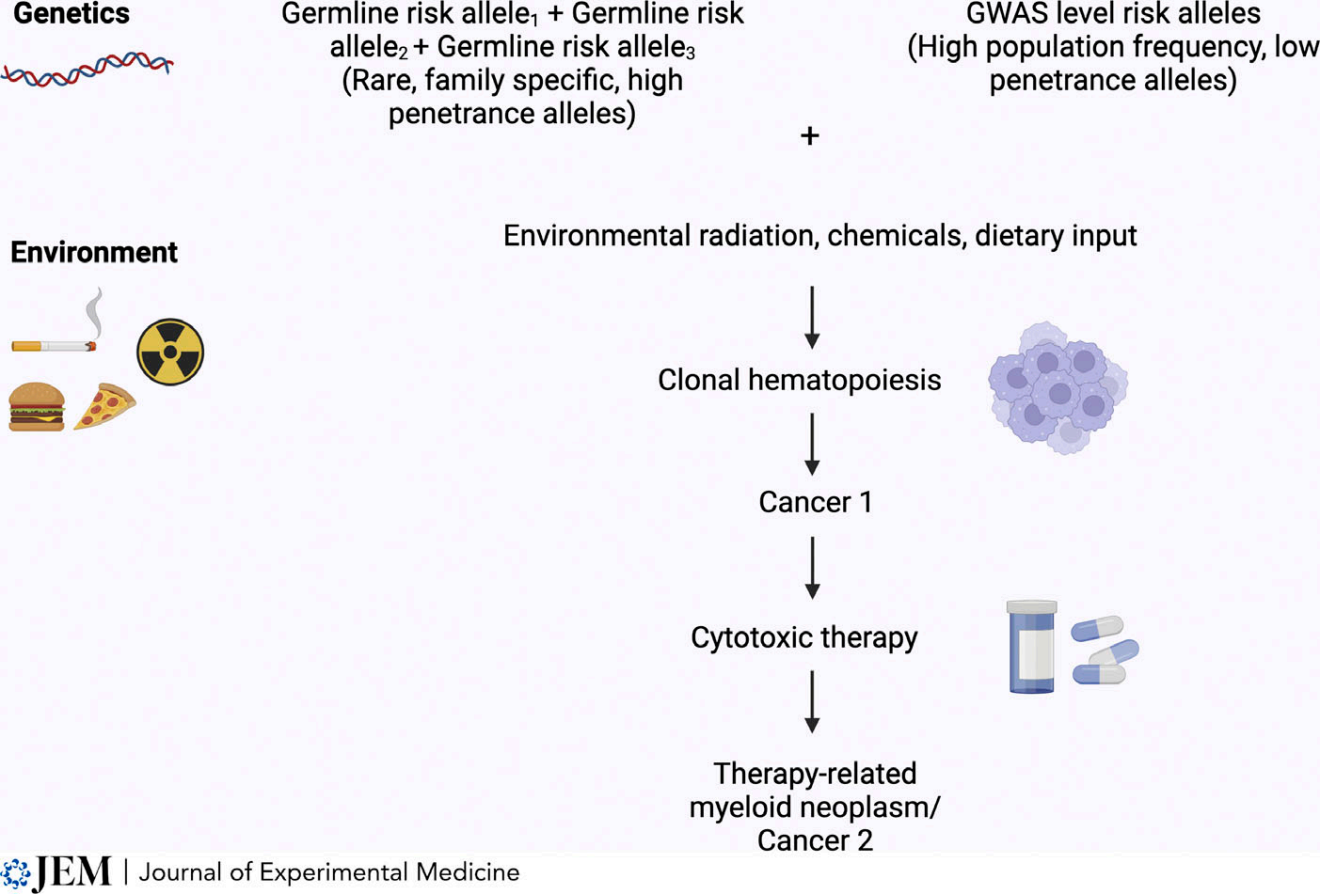
**Cancer risk over an individual’s lifespan.** An individual’s risk for CH and subsequent malignancies is shaped by the combined risk of germline genetic factors, comprised of high population frequency/low penetrance alleles (e.g., found in *TERT*) and low population frequency/high penetrance alleles (e.g., found in *TP53* and *ATM*) with environmental exposures, such as tobacco use, highly processed foods, environmental toxins, and cancer-directed therapies, among others. Created in BioRender. Franco, S. (2024) https://BioRender.com/a12g213.

The interplay between germline genetics and environmental exposures shapes an individual’s risk for CH and subsequent malignancies (refer to Fig. 1). Germline risk can be divided into high population frequency/low penetrance alleles (e.g., *TERT*) and low population frequency/high penetrance alleles (e.g., *TP53* and *ATM*). Kessler et al. conducted a genome-wide association study (GWAS) to identify common, low-penetrance risk alleles associated with the development of CH (Kessler et al., 2022). They identified 24 loci associated with CH, with *TERT* carrying a greater risk compared to other associated gene variants (Kessler et al., 2022). Several other GWAS studies have identified multiple germline variants in the *TERT* locus associated with increased risk of CH (Bick et al., 2020; Dawoud et al., 2020; Kar et al., 2022; Quiros and Vassiliou, 2023; Zink et al., 2017). *TERT* encodes the catalytic subunit of telomerase responsible for maintaining telomerase length (Quiros and Vassiliou, 2023). Although it is transcriptionally silenced in somatic tissues during gestation, expression is maintained in highly proliferative stem cells and is reactivated in 85–90% of malignancies (Quiros and Vassiliou, 2023). This strong association with *TERT* variants and CH suggests that telomeres could play a critical role in the clonal expansion of hematopoietic stem cells with somatic variants (Quiros and Vassiliou, 2023). This intuitively makes sense as preserved telomere length enables HSPCs to divide continuously, resulting in clonal expansion (Vassiliou, 2023). Other germline variants identified by GWAS analysis include: *PARP1*, *SMC4*, *CD164*, *ATM*, *TP53*, *RUNX1*, and *CHEK2* (Vijg and Dong, 2020; Xie et al., 2014). Further analysis of various subtypes of CH based on the involved genes, such as *DNMT3A*, *TET2*, *ASXL1*, *TP53*, *SRSF2*, *JAK2*, and *SF3B1*, revealed that *DNMT3A* is the most common gene associated with CH and has the greatest number of associated risk alleles (*n* = 23); see Table 1 (Kessler et al., 2022).

**Table 1.** Germline variant strength of association with overall CH and various subtypes

	0–0.25		0.26–0.5	0.6–0.75	0.76–1.0	1.1–1.25	1.26–1.5
Overall CH^a^	*ATM*	*MSI2*	*TERT* ^d^		*CHEK2*		
	*BCL2L1*	*ODF3B*			*TET2* ^e^		
	*CD164*	*PARP1*					
	*CNTROB*	*PURB*					
	*DLK1*	*RUNX1*					
	*DNAH2*	*SENP7*					
	*ENPP6*	*SETBP1*					
	*GATA2*	*SMC4*					
	*GSDMC*	*STN1*					
	*HLA-C*	*TERT* ^b^					
	*IL12A*	*TET2* ^c^					
	*ITPR2*	*TP53*					
	*KPNA4*	*TSC22D2*					
	*LY75*	*TYMP*					
	*LY75-CD302*	*ZNF318*					
DNMT3A-CH	*ABCC5*	*MYB*	*FLT3*		*CHEK2* ^f^		*CHEK2* ^g^
	*ATM*	*OBFC*	*TERT*				
	*BCL2L1*	*PARP1*					
	*CD164*	*PURB*					
	*CNTROB*	*RABIF*					
	*DNAH2*	*SENP7*					
	*GSDMC*	*SETBP1*					
	*IL12A*	*SMC4*					
	*ITPR2*	*TCL1A*					
	*KPNA4*	*TET2*					
	*LY75*	*TP53*					
	*LY75-CD302*	*TSC22D2*					
	*MSI2*	*ZNF318*					
TET2-CH	*ATM*		*TCL1A*	*TP53*			
	*GATA2*		*TERT*				
	*THRB*		*THEM209*				
ASXL-CH	*CD164*		*TCL1A*				
TP53-CH							*SEPT3*
JAK2-CH					*JAK2*		

Table displays various the strength of association between multiple germline variant and overall CH as well as various subtypes. This table is adapted from Quiros and Vassiliou (2023).

a Data adapted from Bick et al. (2020); Dawoud et al. (2020); Kar et al. (2022); Kessler et al. (2022); Quiros and Vassiliou (2023); Travaglini et al. (2024).

b Data adapted from Kessler et al. (2022); Travaglini et al. (2024).

c Data adapted from Kessler et al. (2022).

d Data adapted from Bick et al. (2020); Dawoud et al. (2020).

e Data adapted from Kar et al. (2022).

f Data adapted from Kessler et al. (2022).

g Data adapted from Travaglini et al. (2024).

In addition to high population frequency/low penetrance risk alleles, exome-wide association studies have been utilized to identify rare, low population frequency/high penetrance germline variants associated with CH (Kessler et al., 2022). Germline cancer risk alleles in *CHEK2* and *ATM*, as well as in *CTC1* (a gene associated with telomere maintenance and DNA replication), were identified as risk factors for the development of CH via rare variant gene burden testing (Kessler et al., 2022). Although hematopoietic malignancies have not been recognized historically as being associated with inheritable risk, increasing evidence supports the association between deleterious germline variants in *ATM, CHEK2,* and other genes involved in DNA double-strand break repair via homologous recombination repair (HRR) and the development of t-MNs as well as de novo myeloid neoplasms (Franco and Godley, 2024). For example, in patients with ovarian cancer, those with germline variants involved in HRR are 4.3-fold more likely to develop somatic driver variants in genes strongly associated with CH, such as *TP53* and *PPM1D* (Baranwal et al., 2022). This may suggest that patients with deleterious germline variants in HRR genes are predisposed to develop CH due to impaired DNA double-strand break repair, resulting in reliance on less accurate DNA repair mechanisms and increased incidence of somatic variants. This mechanism explains why individuals with *ATM*, *CHEK2*, and other HRR gene variants associated with hereditary breast and ovarian cancer syndromes possess a higher incidence of CH and resulting t-MNs with exposure to cancer-directed therapies.

Importantly, almost all studies of genetic variants associated with CH have been derived from cohorts of individuals of European ancestry (e.g., trans-omics for precision medicine [TOPMEd] and UK Biobank) (Quiros and Vassiliou, 2023). Although the significance of these risk alleles has been validated in combined European/non-European and specific ancestry studies, the paucity of non-European ancestry groups likely limits our ability to identify new risk alleles that exist in non-European populations (Quiros and Vassiliou, 2023).

### Environmental factors

Environmental factors play an equally important role in the development and expansion of CH. Somatic variants in certain genes, such as *PPM1D*, *TP53*, *CHEK2*, and *ASXL1* are particularly susceptible to extrinsic factors (Watson et al., 2020).

#### Age

People acquire somatic variants throughout their lifetime, with an average of at least one pathogenic variant per decade of life (Kusne et al., 2022). Because older individuals have experienced more random mitotic errors over the course of their lifespan and have greater exposure to environmental risk factors, advanced age is the single greatest risk factor for the development of CH (Florez et al., 2022). Both the number of somatic variants and the VAF of individual clones increase with age, resulting in diminished clonal diversity (Kusne et al., 2022). In individuals aged 70 years and older, the prevalence of clones with VAFs >1% is universal, and up to 30–60% of hematopoiesis is composed of expanded clones (Mitchell et al., 2022). In contrast, clones of this size are rarely observed in individuals under age 60 (Joo et al., 2023). Somatic variants in the spliceosome genes *SRSF2* and *SF3B1* are particularly associated with increased age and are almost exclusively found in individuals 70 years and older (Bolton et al., 2020; McKerrell et al., 2015). In contrast, somatic variants in *DNMT3A* tend to occur in younger individuals but expand at a slower rate (Joo et al., 2023). These observed patterns may be reflective of shifting selective pressures on HSPCs over the lifespan, driving various somatic variants to expand at different age ranges (McKerrell et al., 2015).

#### Tobacco

Individuals with CH are more likely to be heavy smokers (Kessler et al., 2022). Similarly, individuals who smoke cigarettes have a 1.2- to 1.5-fold increased risk of CH when compared with non-smokers (Joo et al., 2023). Both the number of CH variants and the VAFs are positively correlated with tobacco use (Bolton et al., 2020). This association appears to be similar for both active and former smokers (Bolton et al., 2020). Compared with other CH variants, somatic variants in *ASXL1* are particularly associated with a history of tobacco use (Kusne et al., 2022).

#### Diet and metabolic disease

Unhealthy lifestyle choices including excessive alcohol use, high intake of red meat, sugar-sweetened beverages, refined grains, high-fat foods, and ultra-processed foods have been associated with increased prevalence of CH (Bhattacharya et al., 2020). The association between metabolic diseases, such as obesity, hypertension, hyperlipidemia, and type 2 diabetes mellitus, with CH remains less clear. Data show decreased rates of CH in women with normal body mass index (BMI) (18.5–25 kg/m^2^) compared to women with obesity (defined as BMI >30 kg/m^2^), whereas other studies show no association between obesity or type 2 diabetes mellitus and CH (Joo et al., 2023). This may be limited by BMI, which is an imperfect predictor of metabolic disease. Interestingly, a higher than genetically predicted BMI, which may be a better indicator of unhealthy lifestyle and metabolic disease, has been associated with significantly increased clone size (Joo et al., 2023).

#### Inflammatory/autoimmune disease

The association between autoimmune disease and increased risk of myeloid neoplasms has been attributed classically to exposure to therapies such as methotrexate and cyclophosphamide. However, studies have shown that the increased risk of myeloid neoplasms is observed even in those without prior treatment exposure (Boddu and Zeidan, 2019). Chronic inflammation and autoimmune diseases appear to result in preferential myeloid differentiation of HSPCs, resulting in decreased heterogeneity, which may contribute to the development of CH (Barreyro et al., 2018). Studies have shown that the prevalence of CH is higher in patients with chronic inflammatory states such as HIV and autoimmune diseases including ulcerative colitis, rheumatoid arthritis, and anti-neutrophil cytoplasmic antibody (ANCA)-associated vasculitis when compared with healthy controls (Kusne et al., 2022). Inflammation appears to be both a driver for and a consequence of CH, resulting in a positive feedback loop (Florez et al., 2022). Although inflammatory states drive the expansion of clonal populations that possess an acquired fitness advantage, CH itself produces a proinflammatory state through dysregulation of innate immune pathways, which have been linked to processes such as atherosclerosis and autoimmune diseases (Florez et al., 2022; Trowbridge and Starczynowski, 2021). Examples of this can be observed in animal models. In mice, *Tet2* loss-of-function results in upregulation of inflammatory cytokines including IL-6, TNF-α, and IL-1β due to impaired intestinal barriers, and *Tet2*^*−/−*^ mice display increased rates of atherosclerosis and colitis compared to wildtype (WT) animals (Kusne et al., 2022).

#### Environmental toxins

Certain occupational and other rare environmental toxins have been shown to increase the risk of CH. Studies conducted on astronauts have revealed an association between CH and exposure to space radiation, with most common variants involving DDR genes, such as *TP53* and *DNMT3A* (Singh and Balasubramanian, 2024). Blood samples from World Trade Center (WTC) first responders revealed significantly increased rates of CH compared to sex, age, and ethnicity non-WTC first responders (odds ratio [OR] 3.14, 95% confidence interval 1.64–6.03, P = 0.0006) (Singh and Balasubramanian, 2024). Although these particular exposures are not applicable to the general population, they emphasize the potential harm of environmental radiation and aerosolized particles on HSPCs.

#### Exposure to cancer-directed therapies

Cytotoxic chemotherapy, external beam radiation, and radionucleotide therapy are strongly associated with the development of CH, whereas exposure to targeted immunotherapy is not (Bolton et al., 2020). Among cytotoxic chemotherapy, topoisomerase II inhibitors and platinum agents are most strongly associated with CH (Bolton et al., 2020). These agents also carry the greatest risk of t-MNs.

Interestingly, the majority of CH variants seen after chemotherapy can be detected even prior to treatment initiation (Kusne et al., 2022). This suggests that cytotoxic therapy most often contributes to CH via positive selection driving clonal expansion of pre-existing somatic variants and is less frequently responsible for acquisition of the original somatic variant (Kusne et al., 2022). Exposure to cytotoxic therapy is specifically associated with CH involving the DDR genes (e.g., *TP53*, *PPM1D*, *ATM*, and *CHEK2*) (Kusne et al., 2022). Retrospective studies have shown that in patients who have received cytotoxic chemotherapy/radiation, clones containing DDR variants outcompeted clones with non-DDR gene variants (Bolton et al., 2020). In contrast, in patients who did not receive interval cancer-directed therapies, non-DDR CH genes outcompeted clones with DDR gene variants (Bolton et al., 2020). This strongly supports the concept that cancer-directed therapies positively select for clones containing somatic variants in DDR genes, such as *TP53*, *PPM1D*, and *CHEK2*, but also that these clones have relatively lower fitness in the absence of exposure to cytotoxic therapy (Bolton et al., 2020).

The rate of CH in patients with cancer also varies based on the type of primary tumor, with the highest rates of CH seen in those with ovarian and thyroid malignancies. The type of CH variants is similar across various malignancies, except that variants in DDR genes (particularly *PPM1D*) are more common in those with ovarian and endometrial cancer (Bolton et al., 2020). Again, this may be because germline variants in HRR genes associated with hereditary endometrial and ovarian cancer, such as *CHEK2* and *ATM*, are associated with increased rates of somatic variants in DDR genes such as *TP53* and *PPM1D* (Baranwal et al., 2022; Franco and Godley, 2024).

## Role of CH in the development of hematopoietic malignancies and solid tumors

### Myeloid neoplasms

The incidence of myeloid neoplasms is high across all CH phenotypes, with an estimated rate of 0.5–1% per year (Kar et al., 2022; Kusne et al., 2022). The risk of transformation increases with the size and number of somatic variants, with clones with VAF >10% conferring the greatest risk of progression to hematopoietic malignancy (Kusne et al., 2022; Quiros and Vassiliou, 2023). One retrospective study found that among patients with CH with VAF >10%, 83% developed AML (OR 6.5, P < 0.001) (Desai et al., 2018). When considering only gene variants associated with myeloid neoplasms, individuals with clones with VAF >10% developed AML in >90% of cases (OR 11.6, P < 0.001) (Desai et al., 2018). In addition to VAF, increased clonal complexity also appears to decrease the latency time between the onset of CH and leukemic transformation (Desai et al., 2018). CCUS (which is associated with a greater number of clones and VAF) carries a greater risk of transformation than CHIP, with an estimated risk of 95% at the 10-year follow-up (Petrone et al., 2023). Lastly, specific gene variants harbor a greater risk of myeloid malignancy. Somatic variants in certain genes, including *TP53*, *U2AF1*, *SRSF2*, *SF3B1*, *IDH1/2*, *TET2*, and *DNMT3A*, are most strongly associated with myeloid transformation (Desai et al., 2018). *TP53* and *U2AF1* variants carry the highest risk of transformation in the setting of extrinsic selective pressures, whereas variants in *DNMT3A* and *TET2* are more common but confer relatively lower risk when found in isolation (Abelson et al., 2018). Notably, *FLT3* and *NPM1*, common driver variants associated with the development of myeloid leukemia, are virtually non-existent in CH, suggesting that the acquisition of these variants likely occurs later in the pathogenesis of AML and is strongly linked to leukemogenesis (Desai et al., 2018; McKerrell et al., 2015). Interestingly, *NPM1* variants frequently co-occur with *DNMT3A* in the pathogenesis of myeloid leukemias (McKerrell et al., 2015). Therefore, it is very plausible that *DNMT3A*, the most common variant associated with CH, often serves as the first hit and leads to clonal expansion with progression to leukemia when accompanied by somatic driver variants in *NPM1* (McKerrell et al., 2015).

### Lymphoid neoplasms

Although much less common, CH is also associated with lymphoid leukemias and lymphomas (Kar et al., 2022). Unlike in the case of myeloid neoplasms, cases of CH associated with lymphoid neoplasms are more evenly distributed across a greater number of gene variants (Niroula et al., 2021). Of note, the increased predisposition to one hematopoietic lineage over the other is not exclusive, and variants in genes commonly associated with CH (e.g., *DNMT3A* and *TET2*) appear to be associated with both myeloid and lymphoid neoplasms (von Beck et al., 2023).

### Solid tumors

The prevalence of CH in patients with solid tumors is between 25% and 30% (Buttigieg and Rauh, 2023). Longitudinal studies have shown that CH (particularly with VAF >10%) increases the risk of solid tumors, including lung, renal, breast, and prostate cancer as well as sarcomas (Buttigieg and Rauh, 2023; Kar et al., 2022). Interestingly, the association between CH and increased predisposition to lung cancer appears to be independent of smoking status and is primarily driven by variants in *DNMT3A* and *ASXL1* (Kessler et al., 2022). As in the case of hematopoietic malignancies, the likelihood of progression from CH increases with a greater number of variants and VAF (Kusne et al., 2022; Quiros and Vassiliou, 2023).

## Role of CH in the development of t-MNs

t-MNs comprise 10–20% of myeloid neoplasms, including MDS, AML, and overlapping MDS/myeloproliferative neoplasms (MDS/MPN) (Baranwal et al., 2022). Because of both patient- and disease-specific characteristics, t-MNs tend to respond poorly to intensive therapy and carry a particularly dismal prognosis, with an average 5-year survival of <10% (Heuser et al., 2016). Compared with de novo myeloid neoplasms, t-MNs carry higher rates of complex karyotype (30–50%), somatic *TP53* variants (15–40%), and abnormalities in chromosomes 5 and 7 (Baranwal et al., 2022; Wong et al., 2014). Previously, it was believed that t-MNs arise purely as a result of the mutagenic effects of cytotoxic therapy. However, it is now recognized that the variants that drive t-MNs often pre-date the initiation of cancer-directed therapies (Bolton et al., 2020). Approximately, 20–60% of patients with t-MNs possess somatic variants in various genes strongly associated with CH, including *DNMT3A*, *TET2*, *ASXL1*, *PPM1D*, and *TP53* (Travaglini et al., 2024). Wong et al. performed whole-genome sequencing in 22 cases of t-MNs and found that the number of somatic SNVs was similar between therapy-related and de novo AML (Wong et al., 2014). Furthermore, in four of seven cases, *TP53* variants were found at low frequencies between 3 and 6 years prior to the development of a t-MN (Wong et al., 2014). These findings indicate that exposure to chemotherapy preferentially selects for expansion of existing somatic variants rather than inducing genome-wide DNA damage (Wong et al., 2014).

As previously discussed, hematopoietic stem cells that possess somatic driver variants display enhanced fitness in the setting of cellular stress due to higher proliferation rates and enhanced avoidance of cell death. Therefore, exposure to cytotoxic therapy positively selects for aggressive clonal populations of cells containing somatic variants in genes such as *TP53*, *PPM1D*, and *CHEK2*, promoting clonal expansion and development of t-MNs (Voso et al., 2021). Interestingly, the therapies that most strongly select for clones containing DDR gene variants (e.g., platinum agents, topoisomerase II inhibitors, and radiation therapy) are the therapies most strongly associated with the development of t-MNs (Bolton et al., 2020). This mechanism likely explains why t-MNs have a higher incidence of *TP53* variants and tend to be more aggressive and less responsive to treatment compared to de novo myeloid neoplasms (Baranwal et al., 2022; Fianchi et al., 2018). This concept is supported by murine models, which have shown that *Tp53*^+/−^ HSPCs preferentially expand with exposure to chemotherapy (Wong et al., 2014). In people, case-control studies have found that among elderly patients with a history of cancer, individuals with CH had a higher risk of developing t-MNs (Gillis et al., 2017). Similarly, those with t-MNs had higher rates of CH-related somatic variants compared with controls (P = 0.024) (Gillis et al., 2017). Bolton et al. concluded that among patients who received chemotherapy, immunotherapy, or radiation therapy, the presence of CH with a VAF ≥2% was associated with an increased risk of t-MNs with a hazard ratio of 6.9 (P < 10^−6^) (Bolton et al., 2020). This risk increased further with a greater number of variants and VAF (Bolton et al., 2020).

In addition to the positive selection of existing clones, the leukemic transformation from CH to t-MNs likely relies on the acquisition of additional somatic variants. Bolton et al. studied 35 cases of t-MN transformation from pre-existing CH and found that 91% of cases were associated with the acquisition of additional somatic variants associated with myeloid neoplasms, including chromosomal aneuploidy and variants in genes such as *FLT3*, *KRAS*, and *NRAS* (Bolton et al., 2020). Among these cases of t-MNs, 40% of patients had variants in *TP53*, the majority of which were present at the time of initial CH testing (Bolton et al., 2020). At the time of leukemic transformation, *TP53* variants co-occurred with isolated chromosomal aneuploidies or complex karyotypes in 92% of cases (Bolton et al., 2020). This once again suggests a mechanism in which individuals with CH who are exposed to cytotoxic therapy have a positive selection of clones containing *TP53* and other DNA damage response (DDR) gene variants, which subsequently attain clonal dominance and acquisition of additional somatic variants, driving the development of aggressive and treatment-resistant t-MNs with higher prevalence of *TP53* variants and complex karyotype.

## Animal models

*TET2* variants often represent an early event in the development of human myeloid malignancies, including AML, MDS, MPNs, and chronic myelomonocytic leukemia (CMML), as well as B- and T-cell neoplasms (Quivoron et al., 2011). *TET2* loss-of-function results in a preleukemic myeloproliferative disorder characterized by myeloproliferation, extramedullary hematopoiesis, and splenomegaly, which may progress to leukemia if additional variants are acquired (Meisel et al., 2018; Quivoron et al., 2011). In in vivo studies, *Tet2*^*−/−*^ mice develop myeloproliferation and myeloid dysplasia that resembles human CMML (Moran-Crusio et al., 2011). *Tet2*^*+/−*^ mice also display increased self-renewal of HSPCs and extramedullary hematopoiesis, suggesting that heterozygous loss-of-function variants also contribute toward malignant transformation (Moran-Crusio et al., 2011).

*Tet2* ^*−/−*^ mice develop various hematopoietic malignancies (73% myeloid, 23% T cell, and 4% B cell) (Zeng et al., 2019). Studies have found that elevated levels of circulating inflammatory cytokines secondary to bacterial translocation promote expansion of myeloid cells and leukemic transformation in *Tet2*^−/−^ mice, likely because *Tet2-*deficient HSPCs experience a selective advantage in times of inflammatory stress (Meisel et al., 2018; Zeng et al., 2019). Elevated proinflammatory such as IL-6, IL-1, and TNF-α are positively correlated with increased myeloid differentiation and expansion in *Tet2*^−/−^ but not WT mice (Meisel et al., 2018; Zeng et al., 2019). This suggests that in the setting of *Tet2* loss-of-function, inflammatory signaling may be associated with the development of myeloid malignancies (Zeng et al., 2019). Because chronic inflammation in *Tet2*^*−/−*^ mice is driven primarily by bacterial translocation secondary to disruption of intestinal barriers, it was theorized that disrupting this process may suppress the subsequent inflammatory response and potentially reduce leukemic transformation (Meisel et al., 2018; Zeng et al., 2019). Meisel et al. found that *Tet2*^*−/−*^ mice raised in germ-free conditions did not develop myeloproliferation and extramedullary hematopoiesis, in contrast to age-matched controls (Meisel et al., 2018). Furthermore, treatment with antibiotics prevented and reversed these changes. In a similar study, Zeng et al. found that *Tet2*^*−/−*^ mice with CD4^+^ T cell lymphoma or CMML that received antibiotics experienced improved survival, decreased spleen size, and reduction in *Tet2*^*−/−*^ tumor cells compared with those that did not receive antibiotics (Zeng et al., 2019). This was in contrast to WT mice, in which there was no difference in outcomes among antibiotic and control groups (Zeng et al., 2019). These findings emphasize the importance of environmental factors, such as the microbiome, which may influence chronic inflammatory states that drive malignant transformation from CH. Furthermore, these findings suggest that suppressing aberrant inflammatory signaling may reduce the risk of malignant transformation in *TET2* CH.

Additional animal models studying common CH variants are summarized in Table 2.

**Table 2.** Animal models studying common CH variants

Animal model	Disease phenotype	CH variant	Citation
Mouse	Hematopoietic	*Tet2*	Ito et al. (2019); Meisel et al. (2018); Zeng et al. (2019)
		*Dnmt3a*	Jeong et al. (2018)
		*Asxl1*	Abdel-Wahab et al. (2013); Hsu et al. (2017); Nagase et al. (2018); Wang et al. (2014)
		*Srsf2*	Kim et al. (2015); Lee et al. (2018)
		*Sf3b1*	Lee et al. (2018); Obeng et al. (2016); Fuster et al. (2017); Sano et al. (2018a, b); Wang et al. (2018)
	Cardiovascular	*Tet2*	Fuster et al. (2017); Sano et al. (2018a, b)
		*Dnmt3a*	Sano et al. (2018a)
		*Jak2*	Wang et al. (2018)
	Inflammatory	*Tet2*	Agrawal et al. (2022); Kim et al. (2021); Miller et al. (2022); Wong et al. (2023)
		*Dnmt3a*	Kim et al. (2021); Wong et al. (2023)

## Opportunities for intervention in individuals with CH

Identification of individuals with CH offers potential opportunities for intervention prior to the development of de novo and therapy-related malignancies; therefore, improved detection and surveillance of CH in the clinic setting is warranted. At present, there are no specific guidelines for the management of pre-malignant CH; however, reasonable interventions include close laboratory monitoring, age-appropriate cancer screening, avoidance of unnecessary radiation, and emphasis on key lifestyle modifications, such as smoking cessation, regular exercise, and healthy/anti-inflammatory diet.

Additional preventative strategies for those with CH are being explored. As previously described, chronic inflammation results in the expansion of clones containing somatic driver mutations that provide a fitness advantage, therefore suppression of aberrant inflammatory signaling could theoretically reduce the risk of malignant transformation in CH. This was observed in *Tet2*^*−/−*^ mice that received antibiotics to decrease proinflammatory signaling from bacterial translocation (Meisel et al., 2018). In humans, prophylactic use of anti-inflammatory drugs for CH has primarily been studied within the context of cardiovascular disease. Analysis of the CANTOS randomized controlled trial showed that the use of canakinumab (an anti–IL-1β monoclonal antibody) in individuals with *TET2*-driven CH resulted in reduced major adverse cardiovascular events and improved anemia of inflammation (Svensson et al., 2022; Woo et al., 2023). An ongoing phase II trial (NCT05641831) is evaluating canakinumab use for the prevention of malignant transformation in individuals with CCUS, with the primary endpoint being time to development of myeloid neoplasm.

Alternative strategies include the use of targeted therapies for high-risk clones, such as selective *TET* and *IDH1/2* inhibitors. As previously described, somatic *TET2* loss-of-function results in myeloid differentiation and clonal expansion with progression to myeloid neoplasms. In these cases, compensatory *TET1/3* activity is needed for the survival of *TET2*-deficient neoplastic hematopoietic stem cells (Guan et al., 2021). Therefore, selective *TET* inhibitors may offer a promising strategy for targeted treatment of *TET2*-associated myeloid neoplasms (Guan et al., 2021). In murine models, *TET* inhibition was found to prevent the expansion of *Tet2*^−/−^ tumor cells effectively (Guan et al., 2021). Beyond myeloid malignancies, selective *TET* inhibition may have a role in *TET2*-associated CH by restricting the expansion of *TET2*-deficient clones, potentially reducing the risk of malignant transformation as well as cardiac co-morbidities (Guan et al., 2021). In addition, ongoing studies are exploring the use of *IDH* inhibitors for patients with somatic *IDH1/2* variants, which carry a relatively high risk of transformation to myeloid malignancy. NCT05030441 and NCT05102370 are ongoing multi-institutional pilot studies looking at the use of *IDH1*-inhibitor ivosidenib and *IDH2*-inhibitor enasidenib for *IDH1-* and *IDH2*-associated CCUS, respectively, to determine whether targeted treatment can decrease the risk of transformation to a hematopoietic malignancy (Petrone et al., 2023).

## Opportunities for intervention in individuals with a primary malignancy

Patients with CH exposed to cytotoxic therapy are at greater risk for the development of t-MNs compared with those without CH. As previously discussed, this is due to positive selection and resulting clonal expansion of pre-existing somatic variants that possess a survival advantage in the face of heightened cellular stress (Travaglini et al., 2024). This suggests a potential benefit of screening for CH in those with solid or hematopoietic malignancies to identify which patients are at greatest risk for the development of t-MNs. With decreases in time and cost of sequencing assays, this may be feasible.

**Figure 2.**
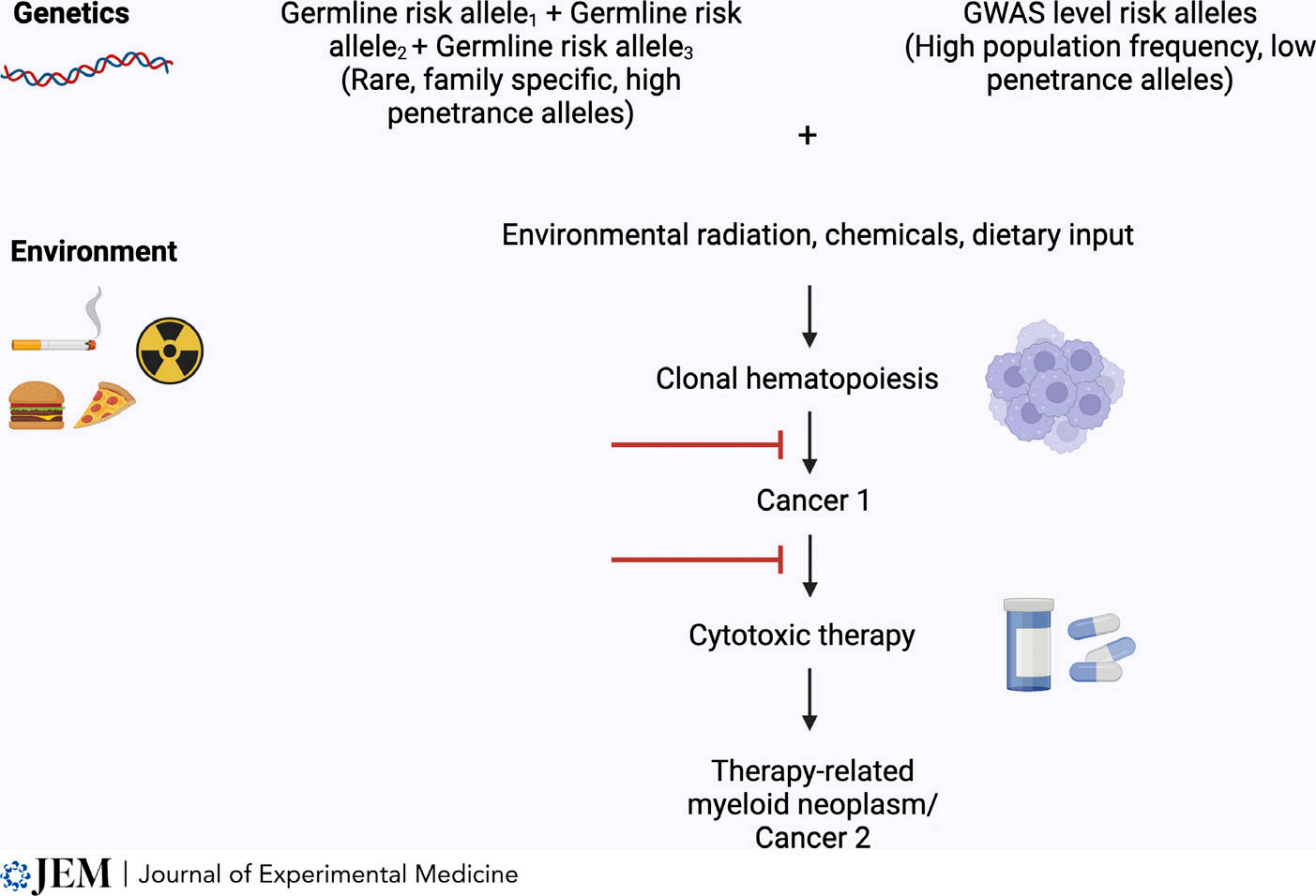
**Points of possible intervention.** There are several points of possible intervention prior to the development of aggressive and often difficult-to-treat malignancies. The first point of possible intervention is in those with premalignant CH and includes improved screening for CH in the outpatient and primary care setting as well as dedicated clinics for laboratory surveillance, age-appropriate cancer screening, and risk reduction via lifestyle modifications. Additionally, further studies are needed regarding the efficacy of anti-inflammatory drugs and targeted therapies for preventing malignant transformation in those with premalignant CH. The second point of possible intervention is in those with a primary malignancy. We argue that CH status should be implemented in treatment decision pathways, such as the use of adjunctive chemotherapy in those with localized disease, as this may help mitigate the risk of future t-MNs in those with underlying CH. Created in BioRender. Franco, S. (2024) https://BioRender.com/a12g213.

Identifying patients with cancer associated with underlying CH may allow for strategies to reduce the risk of potentiating aggressive t-MNs (Fig. 2). For example, knowledge of CH status could influence risk versus benefit decision-making regarding the use of adjuvant systematic chemotherapy in patients with localized disease. According to Bolton et al., 96% of patients with breast cancer have a <1% 10-year absolute risk of subsequent myeloid neoplasm (Bolton et al., 2020). However, for those with the highest risk of developing a t-MN due to the presence of CH, adjuvant chemotherapy in addition to surgical resection increases the risk of developing a t-MN by 9%, exceeding the absolute survival benefit of adjunctive chemotherapy for localized disease (Bolton et al., 2020). Therefore, knowledge of CH status should be taken into account when considering adjunctive chemotherapy for the management of localized disease. Additionally, as previously discussed, certain cancer-directed therapies carry a higher risk of expanding pre-existing clones than others. Cytotoxic chemotherapy (particularly topoisomerase II inhibitors and platinum agents) and radiation therapy are most strongly associated positive selection of existing clones and progression to t-MNs (Bolton et al., 2020). In contrast, immunotherapies do not appear to promote clonal expansion. In one study following patients with MDS treated with chemotherapy versus hematopoietic stem cell transplant (HSCT), the majority of patients had an increased CH number of variants and/or VAF over a span of 9–17 months (Kusne et al., 2022). In a study considering the use of immune checkpoint inhibitors (ICI) for cutaneous melanoma or basal cell carcinoma, the selection and expansion of pre-treatment clonal populations were not observed (Kusne et al., 2022; Miller et al., 2020). This supports the concept that ICI therapy and other forms of immunotherapy may differ from classic cytotoxic therapy in terms of impact on the clonal landscape (Miller et al., 2020). Together, these findings emphasize the need for an individualized approach to treatment in those with cancer and known CH.

Beyond the increased risk for t-MNs with exposure to cytotoxic therapies, the presence of CH can also impact treatment efficacy and toxicity. For example, in autologous stem cell transplantation, the presence of underlying CH is associated with decreased mobilization of stem cells, delayed engraftment, and worse outcomes, including overall survival and relapse-free mortality (Singh and Balasubramanian, 2024). In the context of chimeric antigen receptor (CAR)-T cell therapy, the proinflammatory nature of CH, including elevated levels of circulating IL-1 and IL-6 classically associated with *TET2* variants, may increase the risk of cytokine release syndrome (CRS) and other immune toxicities (Singh and Balasubramanian, 2024). Additionally, deletions of *TET2* and *DNMT3A* have been shown to influence CAR-T function by improving the antitumor effect and preventing T-cell exhaustion (Miller et al., 2021; Singh and Balasubramanian, 2024). Miller et al. conducted a retrospective analysis of 154 patients who underwent CAR-T cell therapy for non-Hodgkin lymphoma (NHL) and multiple myeloma (MM), of which 48% were found to have CH with a VAF >2% (Miller et al., 2021). Among these patients, CH was associated with a higher likelihood of achieving complete remission and an increased rate of CRS, but only in younger individuals (<60 years) (Miller et al., 2021). Saini et al. reported on 114 patients who received anti-CD19 CAR-T cell therapy for large B-cell lymphoma, of which about 37% were found to have CH (Saini et al., 2022). Although they found no difference in rates of CRS or immune effector cell–associated neurotoxicity syndromes (ICANS) among groups, the rate of severe ICANS (grade 3 or higher) was higher in those with CH (45.2% versus 25%, P = 0.038) (Saini et al., 2022). Rates of severe CRS were also higher in those with CH but did not reach statistical significance (Saini et al., 2022). Higher toxicities were most strongly associated with variants in *DNMT3A*, *TET2*, and *AXL1* (Saini et al., 2022). Most recently, Goldsmith et al. conducted a retrospective study of 62 patients who received CAR-T for NHL or MM (Goldsmith et al., 2024). Of these, 24% were found to have at least one pathologic CH variant, with *DNMT3A* being the most common (Goldsmith et al., 2024). Patients with CH were more likely to develop grade 2 or greater CRS compared with those without CH (60% versus 28%, P = 0.023) (Goldsmith et al., 2024). However, there was no difference in rates of ICANS among groups (Goldsmith et al., 2024). Larger, prospective studies are needed to understand the relationship between CH and the efficacy/toxicity of CAR-T cell therapy further.

## Opportunities for intervention in individuals with t-MNs

t-MNs, particularly those with *TP53* variants, often respond poorly to intensive chemotherapy (Travaglini et al., 2024). The current standard of care is hypomethylating agents and venetoclax; however, this strategy does not improve long-term survival (Travaglini et al., 2024). Although allogeneic HSCT is potentially curative, it is rarely an option in these patients due to older age and poor performance status (Travaglini et al., 2024). Therefore, better treatment options are desperately needed. Targeted therapies directed against variants strongly associated with CH, such as *TET* inhibitors, are being explored for pre-malignant CH, but further studies are needed to assess the use of such targeted therapies for t-MNs associated with CH.

As mentioned above, patients with CH may have an enhanced response to T-cell-mediated therapies such as CAR-T cell therapy and bispecific T-cell engagers, particularly those with *DNMT3A* and *TET2* loss-of-function variants, which tend to be proinflammatory and have been associated with greater antitumor activity and decreased T-cell exhaustion. However, CH may also predispose to an increased risk of inflammatory toxicities such as CRS and ICANS. Additional studies are needed.

## Prediction of lifetime cancer risk

Two models currently exist for predicting the risk of progression to hematopoietic malignancy from CH. Weeks et al. sequenced the exomes of 438,890 UK Biobank participants (Weeks et al., 2022). Of the 193,743 participants in which whole-exome sequencing was performed, 11,337 cases of CHIP/CCUS were identified (Weeks et al., 2022). Among this population, 269 (2.37%) developed a myeloid neoplasm over a median follow-up time of 11.7 years (Weeks et al., 2022). Not surprisingly, the rate of transformation to myeloid neoplasm was higher in those with CCUS compared with CHIP, as CCUS is associated with higher VAF and greater clonal complexity (Weeks et al., 2022). Variants involving spliceosome genes (e.g., *SRSF2*, *SF3B1*, and *ZRSR2*) and AML-associated genes (e.g., *IDH1*, *IDH2*, *FLT3*, and *RUNX1*) were associated with increased risk of transformation compared with other CHIP/CCUS variants, carrying a 9.26- and 13.8-fold increased risk, respectively (Weeks et al., 2022). In contrast, single *DNMT3A* variants were associated with a markedly lower risk of malignant transformation (Weeks et al., 2022). Other factors associated with increased risk of progression to myeloid neoplasm included age >65 years, presence of two or more variants, VAF >20% regardless of the variant, elevated mean corpuscular variant, and red cell distribution width, and the presence of cytopenias (Weeks et al., 2022). Using these variables, Weeks and colleagues developed a clonal hematopoiesis risk score to stratify patients into low-, intermediate-, and high-risk groups (available at http://www.chrsapp.com) (Weeks et al., 2022). The majority of cases (87.6%) fell into the low-risk category, 11.3% were found to be intermediate risk and only 1.13% were categorized as high risk (Weeks et al., 2022). The estimated risk of transformation to myeloid neoplasm at 10 years was ∼0.67%, 7.34%, and 50.6% for low-, intermediate-, and high-risk groups, respectively (Weeks et al., 2022). In complementary work, Xie et al. conducted a smaller analysis of 357 patients with CCUS and identified three factors associated with worse prognosis: variants involving spliceosome genes (2 points), platelet count <100 (2.5 points), and the presence of two or more variants (3 points) (Xie et al., 2024). From this, they derived a clonal cytopenia risk scoreto stratify patients into low- (<2.5 points), intermediate- (2.5–4.9 points), and high-risk (≥5 points) categories (Xie et al., 2024). This scoring system predicted the 2-year incidence of myeloid neoplasms as 6.4%, 14.1%, and 37.2% for low-, intermediate-, and high-risk groups, respectively (Xie et al., 2024).

## Future directions

- Recognition of CH as a risk factor for de novo and treatment-related malignancies offers opportunities for intervention prior to the development of aggressive and often difficult-to-treat malignancies. Therefore, there is a need for more widely available screening for CH in the outpatient and primary care setting.
- Along with improved screening, there is a need for dedicated CH clinics for close laboratory surveillance, age-appropriate cancer screening, and risk reduction via lifestyle modifications—particularly in those with high-risk variants (e.g., *TP53* and spliceosome genes), multiple variants, and/or high VAF.
- Further studies are needed to understand the efficacy of anti-inflammatories and targeted therapies in those with premalignant CH better.
- In addition to de novo malignancies, CH significantly increases the risk of t-MNs. Therefore, more routine incorporation of CH status in treatment decision pathways for those with a primary malignancy is needed.
- Additionally, large prospective studies are needed to characterize the impact of CH in the treatment of t-MNs better, including response to targeted treatment options and potentially better efficacy of T-cell-mediated therapies, such as CAR-T cell therapy and bi-specifics. Similarly, we must better understand the potential increased risk of immune toxicity with T-cell-mediated therapies in those with CH and whether CH status can be used as a predictor for immune toxicity.
- We have discussed existing models for predicting the risk of hematopoietic malignancy in those with CH. Using similar principles and incorporation of germline genetics and environmental risk factors, we may be able to predict individual cancer risk over the lifespan.
- Model organisms, like murine models as discussed above, have inherent immunologic and other differences compared with humans. Using humanized murine and other models and incorporating organoid models may render basic scientific studies more relevant and applicable to people.
